# Growth Charts for Tribal, School-Going Children in Jharkhand Using Anthropometry and Lambda-Mu-Sigma Methods to Create Growth Charts

**DOI:** 10.21315/mjms2020.27.4.10

**Published:** 2020-08-19

**Authors:** Dewesh Kumar, Rana Rishabh Kumar, Anit Kujur, Chandramani Kumar, Shalini Sunderam, Vivek Kashyap, Haribansh Kumar Singh

**Affiliations:** 1 Department of Preventive and Social Medicine, Rajendra Institute of Medical Sciences, Ranchi, Jharkhand, India; 2 Department of Preventive and Social Medicine, Patliputra Medical College and Hospital, Dhanbad, Jharkhand, India; 3 Department of Preventive and Social Medicine, Hazaribag Medical College and Hospital, Jharkhand, India

**Keywords:** child health, growth chart, tribal school children

## Abstract

**Background:**

This study intends to find the growth patterns of selected school children. Globally accepted statistical methods were used to evaluate the data and prepare a growth chart.

**Methods:**

This cross-sectional study was conducted with school-going children from 16 selected schools of a tribal district in Jharkhand using multistage cluster random sampling. In each selected school, 60 students, 30 boys and 30 girls, were chosen randomly, totaling 960 children (full data was for 935 children only). Growth charts were created using Lambda-Mu-Sigma (LMS) chart maker version 2.5 for height, weight and body mass index (BMI). In the charts, the LMS values with Z scores for each age and respective height and weight for boys and girls were recorded.

**Results:**

The 468 boys and 467 girls were in the range of 6–14 years of age. Percentile values obtained for the measured heights in centimetres were evaluated and compared with Indian Academy of Pediatrics reference charts for boys and girls for the same age group, and our values were found to be on the lower side. We were able to plot a growth chart of the data set; as the tribal children’s ethnicity is different, this growth chart might be used to assess nutritional status.

**Conclusion:**

We concluded that growth curves for height, weight, and BMI may be used for evaluating children of age 6–14 years in the tribal population. The measures can be a good indicator of their nourishment status and overall growth patterns, which might be indigenous to their ethnicity. A larger sample size of similar tribal populations may give a clearer picture.

## Introduction

Growth charts are integral tools for public health professionals and clinicians alike, providing a way to assess the health status and growth patterns of children and adolescents. Growth charts have evolved over the years in the statistical methods used and the designs of charts (1). Globally, children are evaluated for their well-being on the basis of their nutritional status as evaluated using growth charts and patterns (2). Ever since World Health Organization (WHO) determined growth charts and reference values in 2005 and proposed them as an international growth reference, these have been used in India and adopted by various agencies like the Indian Academy of Pediatrics (IAP) and United Nations Children’s Fund (UNICEF) to evaluate children in India. This was based on the data obtained for children aged 0–5 years on the multicentric growth reference study, a longitudinal, population-based study done on a group of breastfed babies in six countries. The data, thus obtained were merged, and smooth transitions were applied using the Lambda-Mu-Sigma (LMS) method (3). After the charts were available, evidence from various countries presented some altered figures compared to the WHO figures, but speaking in a broad manner, the overall growth patterns across different ethnicities were similar to the growth patterns for the initial three years of life (4–7). However, as ages progressed, the differences in the growth chart became apparent across various groups, remarkably during puberty. Evidence across different populations in terms of height achieved suggest that heights are not similar in children of the same age group, even if they are from the same socioeconomic class and similar geographic locations (8–11). As a population grows over time, the patterns of growth also change, and regular updating of the reference growth charts is mandated to reflect the ongoing growth patterns for the children. These charts can also represent a secular trend due to their updates (12). International and national growth standards do give a brief snapshot about countries where the population is relatively uniform and follows similar dietary patterns; however, in a country like India with varied population heterogeneity along with food diversity, different growth patterns are expected for different population subgroups as they vary significantly from the rest of country due to their own ethnic lifestyles and food habits. The tribal population in Jharkhand has been known to be different than the rest of the country owing to their own indigenous lifestyle, differing food habits due to poor economic conditions, rampant nutritional deprivation and intertribal differences, making it more complex than other tribes in Northeast or Southern India (13–15). Despite recommendations for nutritional assessments of different tribal populations across the country, evidence is sparse, particularly for school children in Jharkhand (16). Studies have repeatedly pointed out the acuteness of malnutrition in the country, which may cause further complications as children continue to grow (17). There is a clear relationship between nourishment and immunity, which implicates further the importance of the health of children who can be exposed to multiple infectious agents during school outings.

A data-based growth chart can serve as a reference for all future studies and a comparative source for understanding the growth and other associated parameters of school children in the tribal region in Jharkhand.

This study intends to determine the growth patterns of the schoolchildren selected by evaluating obtained data and plotting it in graph using the LMS method. The LMS graph thus created will also be used to compare with the IAP growth chart.

## Methods

The present study was conducted in the selected 16 schools under the Gift Milk programme funded by the National Foundation of Nutrition (NFN) India, a joint venture of the National Dairy Development Board (NDDB) and the Rural Electricity Corporation Limited (REC Ltd) (18). The schools were selected randomly from the district of Latehar in Jharkhand as it is a tribal subplan district under Government of India with special emphasis on the tribal population (19). A total of three blocks of the district were chosen for the whole project based on geographic homogeneity, similar demographic populations and logistic availability. Approval for the study was taken by the Institutional Ethical Committee of the Rajendra Institute of Medical Sciences in Ranchi. The project was started in November 2017, and we were to complete the analysis and report-writing by November 2018. We adopted the standard procedures as described in the seminal paper for such projects by Waterlow et al. (20). These guidelines mandate that we have representatives from different age groups and participants from both sexes with a sample size of more than 500 children, use a cross-sectional design, use a defined and reproducible sampling procedure, measure carefully, and, using trained observers, record measurements meticulously using instruments that have been periodically calibrated and tested. In each selected school, 60 students, 30 boys and 30 girls, were chosen randomly, amounting to a total of 960 children between the ages of 6 and 14 years. During data cleaning, owing to some incomplete data on hard copies, we had a final total of 935 children’s records for evaluation. In some schools, we had comparatively fewer children from certain age groups. For example, for age 6, we had only 9 boys and 5 girls, while for age 7 we had 18 children. For older age groups we had even more.

A pilot study was completed initially to check the feasibility of the study and address any problems. All members of the field team were encouraged to participate in the capacity-building. Piloting was done as an external pilot survey in which a nearby, government-run school was selected. Questionnaires and other assessments were used to obtain a full picture of things to come in the field. The piloting was done on 10% of the total sample size of 960 children. The children were selected randomly so that we could a have fair representation of all age groups. We included 10 children from each age group from 6–14 years. Five males and 5 females were randomly selected from each age group. Ten extra children, 5 male and 5 female, were selected, too. The questionnaire was designed after checking its face validity, which was found to be satisfactory by the Principal Investigator. The data entries of 100 children’s data were supervised after 12 trained physicians recorded the measurements of the children during piloting. The test-retest reliability of the questionnaire was found to be satisfactory, with an overall score of *r* = 0.80. All data were collected by the questionnaire designed and finalised in the Department of Preventive and Social Medicine, Rajendra Institute of Medical Sciences Ranchi. The questionnaire had three sections: i) questions related to demographic details; ii) questions related to anthropometric measurements and iii) questions related to dietary habits. Data collected using the anthropometric measurements are used in this paper. We employed the techniques as per the WHO’s monograph (21) to measure all school children. We measured children aged 6–14 years using a stadiometer (Charder HM200PW Wall Mounted Stadiometer), and measurements were performed by one observer following the recommendations. Measurements for height were taken to the nearest 0.1 cm. Freeman’s 15 m fiberglass-top measuring tape was also kept for any need-based measurements. Weight was recorded to the nearest 0.1 kg with minimal clothing. An Omron bathroom scale was used to measure and record the weights. The equipment used was standardised; it was newly purchased for this project and was easily recalibrated, carried and moved around in each school. All data, thus collected were entered in MS Excel sheets and double-checked by trained team members. We also used statistical techniques to minimise the errors expected due to the large volume of data. Data that were collected in the field after piloting were cross-checked by our funders during their field visits. Data after collection in the field on hard copies were entered on MS Office Excel sheets with in-built data checks for all cells in the sheets so that data were not entered in the wrong format. Birth dates were obtained from the children’s school records, which were dependent on the Universal Identity Number UID (Adhaar Card) being mandatorily taken by the schools to cross-check the ages of their enrolled children (22). The decimal age was calculated from their recoded ages and date of visit.

### Inclusion Criterion

After formal consent (permission from district authorities, consent from teachers, consent from adolescents), all randomly selected students were asked about their well-being and willingness to participate. All willing students after random selection were included in the study. Formal consent was also taken from the teachers.

### Exclusion Criterion

Those who were severely ill or not willing to participate were excluded from the study. LMS chart maker version 2.5 was used to create the curves, smooth the height and weight and calculate BMI using the following formula: weight in kg/length or height in m^2^ (23). Data analysis was done using SPSS version 23.0 for calculating percentiles of the obtained values for height, weight and BMI. These were then compared with IAP growth charts.

## Results

While evaluating weight in kg for boys and girls, we found the majority of the boys (377) to be in the age range of 10–13 years; in age group of 6–9 years we had 68 boys, and there were 23 boys in the age group of 14 years. In most of these boys, upon comparing the values of the 3rd, 10th, 25th, 50th, 75th, 90th and 97th percentiles from the IAP, we found these values to be less than that of the IAP chart (Supplementary Table 1). We also saw observed the standard deviation values to be less than that IAP values (Supplementary Table 1). During the evaluation of girl students in different schools, we found most (368) girls to be in the age range of 10–13 years, while in age group of 6–9 years we had only 68 girls. For age group of 14 years, we had 31 girls. Like with the boys, among girl students, when we compared the various percentiles with IAP values, we found our percentile scores to be less than that of the IAP, including the standard deviation scores across girls of age group 6–14 years (Supplementary Table 2). Among boys and girls for age 6 years, as the sample size was smaller, we saw all percentile values obtained during observation to be more than the IAP-recommended percentile values. Percentile values obtained for the measured height in cm were evaluated and compared with the IAP reference chart for the same boys and girls (Supplementary Tables 3 and 4). Here, too, for age 6 in boys and girls, as the sample size was less than 10, observed percentile values were more than the IAP reference chart. However, the number of boys or girls in any age was more than 10, so the percentile values obtained were less than the IAP-recommended values.

**Figure 1:**
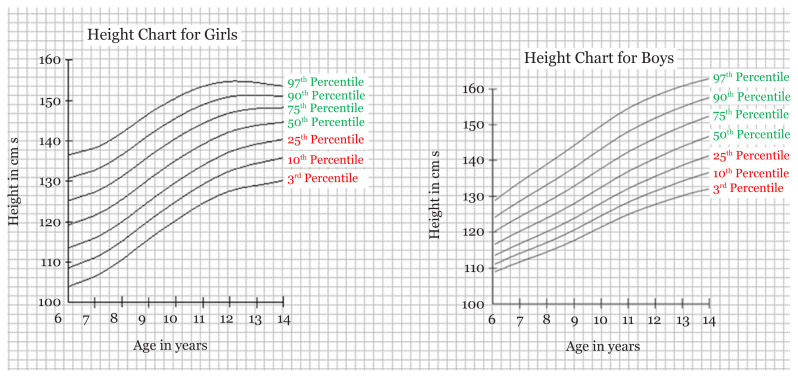
Reference height curve chart for girls and boys with percentile score

**Figure 2:**
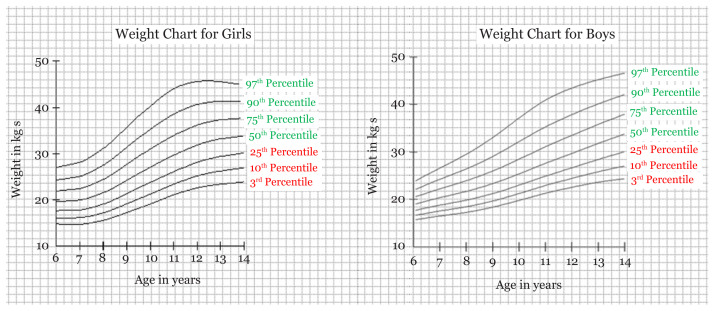
Reference weight curve chart for girls and boys with percentile score

**Figure 3:**
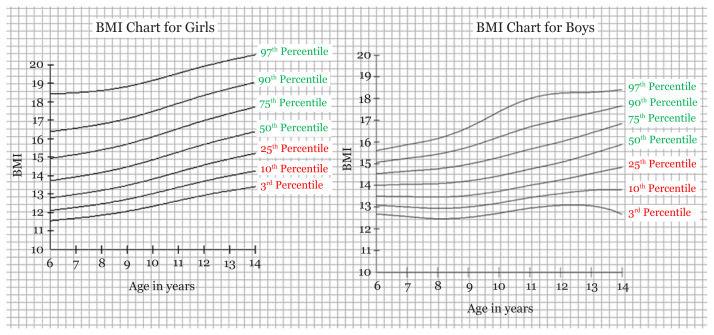
Reference growth curve chart for BMI of girls and boys with percentile score

By using the software LMS chart maker, we were able to have the LMS values with Z scores for each age and their respective height and weight for boys and girls. The M score values were the same as the values of the Z (0 score) (Tables 1 and 2). By calculating BMI from height and weight and using the standard formula for calculating BMI, we were able to have LMS scores of BMI for boys and girls as well. The age range taken in our study was 6–14 years (Tables 1 and 2). By obtaining the values using the software, we were able to make the reference charts/growth curves for weight, height, and BMI for boys and girls aged 6–14 years (Figures 1–3).

## Discussion

Recent evidence suggests wide variations in growth patterns among various populations globally, challenging the notion of using a universal growth chart (24, 25).

In our study, we found the tribal school children population falling behind the growth parameters set by the IAP (percentile values set by the IAP for boys’ and girls’ weight, height and BMI) (Supplementary Tables 1–4) (26). This reflects the importance of prolonged poor nutrition; the WHO and UNICEF define stunting and wasting as two separate, important indicators for nutrition. However, as is evident, different populations can have different charts for measurement, so we need greater sample sizes to determine if the results are due to poor nutrition or the genetics of the tribal populations specific to Jharkhand. We also propose the Z scores using the LMS method with value of median (M) as the value of Z score 0, along with Z scores of −1, −2, −3 and +1, +2, +3 (Figures 1–3). Based on the results thus obtained, we propose the growth curves with percentile values of the 3rd, 10th, 25th, 50th, 75th, 90th and 97th percentiles. We have not compared our values to those of international standards like those of the WHO or the Centres for Disease Control and Prevention (CDC) as the recent IAP norms have been re-evaluated to conform to international standards.

In similar studies done elsewhere, the growth charts have shown a higher curve when compared to WHO curves for similar percentile values. In our study, we also found that if the number of students was less than 10 or 20, the percentile values were on par with the IAP charts for both sexes (Supplementary Tables 3 and 4), especially for girls. This can be attributed to the benefits of the existing mid-day-meal programme and the overall better attendance of girls as visualised and documented by our team during our visits to the schools. Evidence elsewhere has also suggested benefits in the overall nutrition of the mid-day-meal scheme in India (30).

## Conclusion

We believe that the present study is sufficient to be projected as a template for school-going children in Jharkhand owing to our sampling procedure following the multi-stage probability random sampling method, which is considered to be one of the strongest methods for studying a sample in large population. This coupled with the reduction of intra- and inter-observation biases, employing accurate instruments and having trained medical professionals taking measurements makes this study better in the sense of quality. Considering this, we recommend our growth curves of height, weight and BMI to be used when studies are conducted to evaluate children of the age group 6–14 years for the tribal population residing in the state of Jharkhand.

## Supplementary File

The comparative values of the percentile scores obtained against the Indian Academy of Pediatrics (IAP) recommended percentile values

**Supplementary Table 1:** Boys’ weight in kg with percentile values

	Age	*n*	3rd	10th	25th	50th	75th	90th	97th	mean ± SD
Observed	6	9	18.4	18.4	19.1	19.8	21.2			20.4±2.66
IAP	6		14.5	15.8	17.4	19.3	21.7	24.6	28.3	±3.6
Observed	7	8	18.4	18.4	19.52	20.45	22.45			21.7±3.8
IAP	7		16	17.6	19.6	21.9	24.9	28.6	33.4	±4.2
Observed	8	16	14.6	16.98	18.87	21.75	23.1	26.72		21.43±3.37
IAP	8		17.5	19.5	21.9	24.8	28.5	33.2	39.4	±5.7
Observed	9	35	18.08	20.06	20.8	23.6	25.9	27.64	33.86	23.78±3.49
IAP	9		19.1	21.5	24.3	27.9	32.3	38	45.5	±6.3
Observed	10	80	20.17	21.4	23.02	25.65	29.2	34.49	45.26	27.00±5.73
IAP	10		20.7	23.5	26.9	31.1	36.3	43	51.8	±7.9
Observed	11	146	20.98	22.2	25.1	28.3	32	39.13	44.19	29.44±5.99
IAP	11		22.6	25.9	29.8	34.7	40.9	48.7	58.7	±8.9
Observed	12	106	22.3	24.6	27.07	30.2	34.55	39.76	43.69	31.26±5.66
IAP	12		24.9	28.7	33.3	39	46	54.8	66.1	±10.0
Observed	13	45	20.81	24.6	27.95	34.4	39.55	43.92	50.7	33.94±7.40
IAP	13		27.5	31.8	37	43.3	51.1	60.7	72.6	±11.3
Observed	14	23	22.8	25.26	31.8	34.1	37.2	41.58		34.09±5.38
IAP	14		30.7	35.5	41.3	48.2	56.4	66.3	78.3	±12.1

Note: Red coloured entries show values observed to be less than the IAP growth chart values

**Supplementary Table 2:** Girls’ weight in kg with percentile values

	Age	*n*	3rd	10th	25th	50th	75th	90th	97th	mean ± SD
Observed	6	5	15.7	15.7	17.05	20.1	24			20.4±3.80
IAP	6		13.7	15.1	16.7	18.7	21.3	24.6	29.1	±9.1
Observed	7	10	15.6	15.63	16.72	17.75	21.4	23.5		18.8±3.81
IAP	7		15.1	16.8	18.7	21.2	24.2	28.2	33.4	±3.4
Observed	8	14	15.8	15.95	18.8	22.95	25.77	27.5		22.22±4.06
IAP	8		16.7	18.7	21.1	24	27.6	32.2	38.1	±8.1
Observed	9	39	18.34	19.5	21.1	23.7	26.4	31.6	38.68	24.40±4.61
IAP	9		18.5	20.9	23.7	27.2	31.5	36.7	43.4	±3.4
Observed	10	90	18.67	21.03	23.27	26.7	31.55	37.39	39.78	27.74±6.0
IAP	10		20.7	23.5	26.9	31	36	42	49.4	±9.4
Observed	11	102	22.31	24.26	25.8	28.75	31.72	37.78	43.41	29.81±5.99
IAP	11		23.3	26.7	30.7	35.4	41	47.7	55.9	±5.9
Observed	12	122	23.1	24.93	26.95	30.3	35.45	40.63	44.48	31.62±5.90
IAP	12		26.2	30	34.5	39.8	46	53.4	62.1	±2.1
Observed	13	54	24.22	25.8	28.95	32.6	37.1	39.45	42.24	32.78±4.99
IAP	13		28.9	33.1	37.9	43.6	50.2	57.9	67.1	±7.1
Observed	14	31	24.1	27.44	28.6	34	36.3	42.28		33.77±5.14
IAP	14		31.3	35.6	40.6	46.4	53.2	61.1	70.4	±0.4

Note: Red coloured entries show values observed to be less than the IAP growth chart values

**Supplementary Table 3:** Boys’ height in cm with percentile values

	Age	*n*	3rd	10th	25th	50th	75th	90th	97th	mean ± SD
Observed	6	9	113	113	117.5	120.5	124			121.44±6.41
IAP	6		104.2	107.7	111.2	114.8	118.5	122.2	126	±
Observed	7	8	115	115	118.25	121.75	126.37			123±7.11
IAP	7		109.3	113	116.8	120.7	124.6	128.6	132.6	±
Observed	8	16	111	114.5	117.125	122.75	129.5	135		123.4±7.167
IAP	8		114.3	118.2	122.3	126.4	130.5	134.8	139.1	±39.
Observed	9	35	117.08	121	123.5	126.9	132	139.4	145.32	128.4±6.84
IAP	9		119	123.2	127.5	131.8	136.3	140.7	145.3	±45.
Observed	10	80	121	125	129	133	139.37	143.97	151.85	133.92±7.57
IAP	10		123.6	128.1	132.6	137.2	141.9	146.6	151.4	±51.
Observed	11	146	123	127.94	132.37	138.5	142.52	147.8	153.01	138.155±8.03
IAP	11		128.2	133	137.8	142.7	147.6	152.5	157.5	±57.
Observed	12	106	126.31	131	135.37	141	145.15	150.8	157.79	140.98±7.76
IAP	12		133.2	138.3	143.3	148.4	153.5	158.6	163.7	±8.1
Observed	13	45	124.31	130.92	136.5	145	152	155	159.17	143.82±9.21
IAP	13		138.3	143.7	149	154.3	159.5	164.7	169.9	±69.
Observed	14	23	134	137.02	139	145.5	151.7	157.18		145.67±7.26
IAP	14		143.4	149	154.5	159.9	165.1	170.3	175.4	±75.

Note: Red coloured entries show values observed to be less than the IAP growth chart values

**Supplementary Table 4:** Girls’ height in cm with percentile values

	Age	*n*	3rd	10th	25th	50th	75th	90th	97th	mean ± SD
Observed	6	5	109	109	113	120.5	130.75			121.80±9.81
IAP	6		102.3	106	109.7	113.5	117.4	121.5	125.6	±25.
Observed	7	10	106	106.3	109.75	119.5	123.62	127.45		117.30±7.764
IAP	7		107.4	111.4	115.4	119.4	123.5	127.7	131.9	±31.
Observed	8	14	112	113	115	127	135	136		125.66±8.85
IAP	8		112.6	116.8	121.1	125.4	129.6	133.9	138.1	±38.
Observed	9	39	120.64	122	124	129.7	136	143	148.76	130.76±7.41
IAP	9		117.8	122.4	126.9	131.4	135.8	140.2	144.5	±44.
Observed	10	90	118.19	123.55	128	134.5	142.2	147.9	150.63	134.83±8.66
IAP	10		123.3	128.1	132.8	137.4	142	146.4	150.8	±50.
Observed	11	102	127.48	130.36	133.6	138.5	143.12	148.88	156.45	139.13±7.76
IAP	11		128.8	133.7	138.6	143.3	147.9	152.4	156.8	±56.
Observed	12	122	128.51	133.01	136.01	141.85	148.11	152.63	157.65	142.08±7.53
IAP	12		134	138.9	143.7	148.4	153	157.5	162	±62.
Observed	13	54	128.25	135	138.87	143.8	148.12	154.5	158.7	143.84±7.22
IAP	13		138.2	142.9	147.6	152.2	156.8	161.3	165.9	±65.
Observed	14	31	129	136.81	142.01	146	150.6	152.9		145.42±6.11
IAP	14		141.3	145.8	150.2	154.7	159.2	163.7	168.2	±68.

Note: Red coloured entries show values observed to be less than the IAP growth chart values

## Figures and Tables

**Table 1:** LMS values and Z scores for boys’ and girls’ weight (kg) (values of M and Z score of 0 are same)

LMS values and Z scores for boy’s weight (kg)									

Age	L	M	S	−3	−2	−1	+1	+2	+3
6	−1.14555	19.72371	0.109339	16.39805	17.32013	18.3756	21.3035	22.97853	24.9362
7	−1.15034	20.87505	0.125019	16.95297	18.02042	19.26084	22.81126	24.9194	27.45955
8	−1.18715	21.32745	0.138624	16.99377	18.1523	19.51784	23.54948	26.037	29.13597
9	−1.31613	23.06292	0.149187	18.16369	19.44694	20.98334	25.69169	28.74495	32.73445
10	−1.41934	25.3406	0.157877	19.77924	21.21155	22.94821	28.4417	32.16594	37.26144
11	−1.3428	27.76437	0.164005	21.44869	23.07194	25.04358	31.29647	35.54165	41.34731
12	−1.03552	29.51492	0.168009	22.45525	24.3012	26.51595	33.29594	37.65652	43.27733
13	−0.48786	31.93998	0.170266	23.71968	25.95865	28.56316	35.94692	40.2284	45.24367
14	−0.20389	33.90303	0.17157	24.28141	27.07328	30.15633	38.01126	42.03885	46.34071

**LMS values and Z scores for girl’s weight (kg)**									

6	−0.51297	19.59918	0.159398	14.82959	16.1387	17.65176	21.89106	24.31952	27.14001
7	−0.48981	19.89915	0.171595	14.7471	16.14848	17.78046	22.41715	25.1122	28.27487
8	−0.46599	21.52224	0.183053	15.63921	17.22659	19.08764	24.44066	27.59181	31.32291
9	−0.43749	24.2	0.192335	17.297	19.14939	21.33123	27.65779	31.41137	35.87892
10	−0.38446	27.09217	0.197152	19.15574	21.28739	23.79691	31.05443	35.34066	40.4187
11	−0.30959	29.66316	0.194643	20.97062	23.32729	26.08131	33.91756	38.45578	43.74809
12	−9.1602	31.87909	0.186929	22.55412	25.15312	28.12309	36.18937	40.61659	45.57276
13	0.209076	33.1694	0.177235	23.47885	26.28299	29.38738	37.32641	41.41051	45.78088
14	0.50894	33.80515	0.165311	24.0985	27.01638	30.13911	37.67756	41.339	45.11665

**Table 2:** LMS values and Z scores for girls’ and boys’ height (kg) (values of M and Z score of 0 are same)

LMS values and Z scores for boys’ height (cm)									

Age	L	M	S	−3	−2	−1	+1	+2	+3
6	−3.98546	118.315	4.53E-02	109.9464	112.2945	114.9521	122.2337	126.3915	131.2957
7	−3.73893	121.0166	4.81E-02	111.9415	114.4843	117.3658	125.277	129.8042	135.1521
8	−3.51546	123.8385	5.06E-02	114.0858	116.8174	119.914	128.4191	133.2859	139.0322
9	−3.3768	127.7022	5.24E-02	117.2813	120.1977	123.5061	132.6037	137.8159	143.9748
10	−3.15496	132.5033	5.41E-02	121.3155	124.4532	128.0067	137.7387	143.2819	149.7946
11	−2.69808	137.0053	0.05533	124.9984	128.3983	132.2193	142.5018	148.2238	154.8061
12	−1.94E+00	140.5078	0.055834	127.7023	131.4027	135.4927	146.1117	151.7612	158.0194
13	−0.83078	143.8489	5.52E-02	130.2141	134.2929	138.6699	149.3933	154.7279	160.3461
14	0.431266	146.7704	5.37E-02	132.3812	136.8742	141.513	152.1372	157.0614	162.0098

**LMS values and Z scores for girls’ height (cm)**									

6	−3.98546	118.315	4.53E-02	109.9464	112.2945	114.9521	122.2337	126.3915	131.2957
7	−3.73893	121.0166	4.81E-02	111.9415	114.4843	117.3658	125.277	129.8042	135.1521
8	−3.51546	123.8385	5.06E-02	114.0858	116.8174	119.914	128.4191	133.2859	139.0322
9	−3.3768	127.7022	5.24E-02	117.2813	120.1977	123.5061	132.6037	137.8159	143.9748
10	−3.15496	132.5033	5.41E-02	121.3155	124.4532	128.0067	137.7387	143.2819	149.7946
11	−2.69808	137.0053	0.05533	124.9984	128.3983	132.2193	142.5018	148.2238	154.8061
12	−1.94E+00	140.5078	0.055834	127.7023	131.4027	135.4927	146.1117	151.7612	158.0194
13	−0.83078	143.8489	5.52E-02	130.2141	134.2929	138.6699	149.3933	154.7279	160.3461
14	−0.431266	146.7704	5.37E-02	132.3812	136.8742	141.513	152.1372	157.0614	162.0098
